# Complete Genome Assembly of Yersinia pseudotuberculosis IP2666pIB1

**DOI:** 10.1128/MRA.01592-18

**Published:** 2019-02-14

**Authors:** Adam Zoubeidi, Leah Schwiesow, Victoria Auerbuch, Hanh N. Lam

**Affiliations:** a Department of Microbiology and Environmental Toxicology, University of California, Santa Cruz, California, USA; b Department of Molecular, Cell, and Developmental Biology, University of California, Santa Cruz, California, USA; University of Maryland School of Medicine

## Abstract

Yersinia pseudotuberculosis, closely related to Yersinia pestis, is a human pathogen and model organism for studying bacterial pathogenesis. To aid in genomic analysis and understanding bacterial virulence, we sequenced and assembled the complete genome of the human pathogen Yersinia pseudotuberculosis IP2666pIB1.

## ANNOUNCEMENT

Three species within the *Yersinia* genus are human pathogens, Yersinia pestis, Yersinia pseudotuberculosis, and Yersinia enterocolitica. All three pathogenic *Yersinia* species harbor a 70-kb virulence plasmid referred to as pYV, which encodes a type III secretion system critical for virulence (1 – 5). Three mouse virulent Y. pseudotuberculosis strains, YPIII, IP32953, and IP2666, are commonly used for analysis of Y. pseudotuberculosis pathogenicity. The IP2666pIB1 strain, which has been used as the basis for a number of studies (6 – 8), was generated by the Bliska lab (9) by curing the IP2666 strain of its native virulence plasmid and inserting the well-characterized pYV virulence plasmid from YPIII, called pIB1. Although many bacterial genome sequences were released, the IP2666pIB1 genome sequence was not available. Here, we present a complete sequence of the chromosome and pIB1 plasmid of Y. pseudotuberculosis IP2666pIB1.

Y. pseudotuberculosis IP2666pIB1 was grown in 2xYT (yeast extract-tryptone) at 26°C, shaken overnight. The culture was diluted to an optical density (OD_600_) of 0.1 and grown at 26°C, and cells were pelleted when the culture reached an OD_600_ of 0.8. Genomic DNA was extracted with a DNeasy blood and tissue kit (Qiagen). The samples were sent to the DNA Technologies Core at the University of California, Davis, for library preparation with the DNA sequencing kit 4.0 v2 with C4 chemistry, PacBio RS II sequencing (library preparation followed by size selection of 15 kb with Blue Pippin), and MiSeq paired-end sequencing with a 300-bp read length.

Trimmomatic version 0.36 (10) was used to trim off low-quality bases and adapter sequences from MiSeq reads. The trimmed, paired-end reads were used to assemble the genome. PacBio sequences were trimmed with Canu 1.7 (11) during assembly. The average PacBio sequence length is 20 kb. The genome sequence was assembled from two data sets from PacBio long reads (161,000 reads) and MiSeq paired-end reads (2,956,000 reads), resulting in more than 50× coverage. The two data sets were assembled together with SPAdes v3.11.1 (12), and PacBio reads were assembled with Canu 1.7 using default parameters (11). Outputs from the two programs were aligned and visualized in SeaView v4.5.2 (13). The assembled genome was manually inspected and curated in Artemis 16.0.0 (14) to a high-quality completion (15). Briefly, at positions where there were differences between the two assembled sequences aligned and visualized in SeaView, the high-quality sequence reads aligned to the genome were inspected in Artemis to reconcile the disagreements. The genome sequence was annotated with the NCBI Prokaryotic Genome Annotation Pipeline (PGAP) (16, 17).

The chromosome size is 4,614,856 bp, with 47.5% GC content, 4,115 predicted coding sequences, 102 ribosomal and transfer RNAs, and 182 pseudogenes. The pIB1 virulence plasmid showed 44.8% GC content and 96 coding sequences. It is important to note that, unlike Y. pestis CO92 (GenBank accession number NC_003143) and Y. pseudotuberculosis IP32953 (GenBank accession number NZ_CP009712), the entire high-pathogenicity island on the *pgm* locus (18) containing yersiniabactin biosynthetic genes is absent in this strain, which is similar to Y. pseudotuberculosis YPIII (GenBank accession number CP009792) (19).

### Data availability.

The Yersinia pseudotuberculosis IP2666pIB1 project has been deposited in the National Center of Biotechnology Information (NCBI) under the accession numbers CP032566 and CP032567 (BioProject number PRJNA475632). The raw sequencing reads have also been submitted to the Sequence Read Archive (SRA) under accession numbers SRR8061175, SRR8061176, and SRR8061177.
